# Life Satisfaction Factors, Stress, and Depressive Symptoms among Young Women Living in Urban Kampala: Findings from the TOPOWA Project Pilot Studies

**DOI:** 10.3390/ijerph21020184

**Published:** 2024-02-06

**Authors:** Rachel E. Culbreth, Karen E. Nielsen, Kate Mobley, Jane Palmier, Paul Bukuluki, Monica H. Swahn

**Affiliations:** 1 American College of Medical Toxicology, 10645 N. Tatum Blvd, Phoenix, AZ 85028, USA; rachel.culbreth@acmt.net; 2 School of Public Health, Georgia State University, 140 Decatur Street, Atlanta, GA 30303, USA; knielsen4@gsu.edu; 3 School of Data Science and Analytics, College of Computing and Software Engineering, Kennesaw State University, Kennesaw, GA 30144, USA; kmoble23@students.kennesaw.edu; 4 Wellstar College of Health and Human Services, Kennesaw State University, Kennesaw, GA 30144, USA; jpalmier@kennesaw.edu; 5 School of Social Sciences, Makerere University, Kampala P.O. Box 7062, Uganda; paul.bukuluki@mak.ac.ug

**Keywords:** mental health, young women, social determinants of health, urban health

## Abstract

Young women living in Kampala, Uganda, often face adversities related to Social Determinants of Mental Health (SDoMH) including poverty, food scarcity, environmental stressors such as high levels of community violence, and lack of adequate healthcare access. Two consecutive pilot studies were conducted to assess the feasibility and acceptability of survey questions, wearable fitness trackers, and daily diaries before launching a larger prospective cohort study. Preliminary associations between SDoMH factors with depressive symptoms, stress levels, and life satisfaction were examined among the total sample of 60 women across two pilot studies. A total of 32.2% of respondents (out of *n* = 59) reported being depressed most or all of the time in the past 30 days. Frequent depressive symptoms correlated with food insecurity ($\chi^{2}$ = 5.38, *df* = 1, *p* = 0.02). Higher stress levels were significantly associated with lower overall life satisfaction scores (*t* = 2.74, *df* = 6.20, *p* = 0.03). Additionally, frequent depressive symptoms were associated with lower satisfaction scores in the living conditions and lifestyle domain (*t* = 2.22, *df* = 36.18, *p* = 0.03). However, overall life satisfaction scores and other domains (social relationships and personal independence) were not statistically associated with frequent depressive symptoms. Identifying the most impactful SDoMH factors among young women in Kampala can inform targeted approaches to improve mental health outcomes.

## 1. Introduction

Three-quarters of the global mental health disease burden are in low- and middle-income countries (LMICs) [1]. Mental health concerns, including depression and anxiety, are increasing in developing countries, particularly among women [2,3]. Young women living in Uganda (specifically the slums of Kampala) often face additional adversities described as Social Determinants of Mental Health (SDoMH) [4,5], such as poverty, food scarcity, environmental stressors, lack of healthcare access, barriers to reproductive care, and inadequate job opportunities that may further increase their risk of developing depression and poor mental health outcomes [6,7]. However, the majority of research on depression and mental health to date is conducted in high-income settings [3], creating an urgent need to address the research and data needs in low-resource settings and better contextualize the SDoMH. 

Low-resource settings, including areas with high levels of poverty, have complex, multi-level factors associated with mental illness [4,5,8,9]. The SDoMH framework posits that the structural and community-level inequities that affect disadvantaged individuals perpetuate mental illness risks across the lifespan. These structural inequities include policies, access to healthcare, economic and political factors, and cultural norms and often affect multiple generations. Individual and family-level factors such as food insecurity and poor housing conditions, which may additionally be downstream factors from structural and political inequities, are also linked to poor mental health outcomes [4].

An estimated one in four to one in three persons in Uganda is affected by depression or anxiety disorders [10,11]. A meta-analysis showed that 30.2% of Ugandan adults report depression [11]. Depression severity also increases the risk for suicidal behaviors [12]. Suicidal behavior is strongly associated with increased stress from financial constraints [12], which was recently further exacerbated in Uganda during the COVID-19 lockdowns and other public health restriction measures [13]. Other factors such as water insecurity has also been independently linked to more severe depressive symptoms among Ugandan refugees, even after adjusting for social support and food insecurity [14]. These findings are consistent with the SDoMH framework [5] and prior work linking poverty to depression and anxiety [9]. Another way to understand the role of these social drivers is through Maslow’s Hierarchy of Needs (MHN), where individuals report higher motivation and life satisfaction when basic needs are met [15]. For example, the MHN framework states that the basic needs of life (e.g., air, water, food, shelter, clothing, and sleep) are essential before achieving life satisfaction associated with self-esteem, love and belonging, and self-actualization [15]. 

While some of the associations pertaining to economic insecurity, life satisfaction, and depression have largely been studied before [16,17], it is unclear how they present specifically for young women who are living in the slums of Kampala, Uganda, who represent a unique and underserved population. Slums are informal settlements that are characterized by poor housing conditions and a lack of adequate sanitation [18,19,20]. While several studies have examined mental health outcomes among individuals living in slum communities [6,7], a literature gap exists specifically for women and young girls, who are faced with high levels of sex work, gender-based violence, and problem drinking and related harms [6,7,21]. 

Given the complex adversities these women face within the urban environment, understanding the SDoMH framework is imperative for informing interventions in low-resource settings. Utilizing findings from a pilot study of young women living in the slums of Kampala, the objectives of this analysis are to (1) determine the differences in demographic characteristics and economic support between those with frequent depressive symptoms compared to those with less frequent depressive symptoms who live in urban slums, (2) examine the differences in demographic characteristics and economic support between those with low, moderate, and high perceived stress levels among those who live in the urban slums, and (3) determine the quality-of-life (QOL) factors across three domains (lifestyle and living conditions, social relationships, and personal independence) associated with higher stress levels and more frequent depressive symptoms among those who live in urban slums. The results from this study are intended to inform intervention targets for improving mental health symptoms among youth in urban environments in low-resource settings and to examine the complex relationships between financial distress and SDoMH in low-resource settings.

## 2. Materials and Methods

### 2.1. Participant Recruitment

This study consists of data obtained from two consecutive pilot studies conducted across three neighborhoods (Banda, Bwaise, and Makindye) among young women and girls in the slums of Kampala, Uganda. These two pilot studies preceded the implementation of a larger longitudinal cohort study called TOPOWA (meaning empowerment). The objectives of these pilot studies were to assess the feasibility and acceptability of (1) daily diaries to measure activities and sleep health, (2) wearable fitness trackers to objectively measure activities and sleep health, and (3) survey questions for the subsequent longitudinal study. 

Participants were recruited from the Uganda Youth Development Link (UYDEL) drop-in centers. UYDEL operates 13 drop-in centers across Uganda where youth can receive psychosocial and vocational services. Three centers, located in the Banda, Bwaise, and Makindye neighborhoods in and around Kampala, were selected for participant recruitment. The UYDEL centers in these three neighborhoods were chosen because they had the largest enrollment of adolescent girls and young women 18–24 years of age (inclusion criteria) and had the capacity to accommodate the study protocol. These three UYDEL centers are also located in close proximity to densely populated slum communities, where participants for both the intervention and control groups will be recruited for the larger TOPOWA (meaning empowerment) project. Because study participants were recruited from within a 2000 m radius of the UYDEL center, it was important that the chosen UYDEL centers be situated near slum communities from which participants could be recruited.

For the current pilot studies, a total of 60 women (*n* = 30 from each pilot study) ages 18–24 were recruited from the three neighborhoods, resulting in 20 women in total from each site across the two pilot studies. During the initial planned pilot study, a few challenges arose including confusion around wearable fitness trackers and capabilities, and a resulting inconsistency in the utilization of the devices. Therefore, a second pilot study that included providing participants with additional information about the wearable fitness trackers, including assurances that they were waterproof and did not record audio, was implemented approximately 4 months later. The findings from both pilot studies informed the finalization of protocols for the larger prospective cohort study. In addition to testing the feasibility and acceptability of the wearable fitness trackers and daily diaries, participants were asked to complete a baseline survey. The survey questions remained consistent across the two pilot studies.

### 2.2. TOPOWA Project 

The broader National Institutes of Mental Health (NIMH)-funded TOPOWA project is comprised of several qualitative studies including focus groups and a photovoice project as well as the main TOPOWA cohort study, all designed with a shared goal of understanding the SDoMH among adolescent girls and young women living in the slums of Kampala. The main cohort study consists of 300 women who are enrolled into an intervention cohort (N = 150) and a community cohort (N = 150), recruited from the same three neighborhoods as the pilot studies (Banda, Bwaise, and Makindye). The intervention cohort will consist of women who are receiving vocational and empowerment training (socioeconomic strengthening targeted training) through Uganda Youth Development Link (UYDEL). As part of the vocational training, UYDEL provides start-up kits, business skills, and internship placements. Participants will also have access to other UYDEL services, including counseling, linkage to care for sexually transmitted infections and HIV, and community and social support from the providers and other women participating in UYDEL. The community cohort will not receive this training. This longitudinal, observational study will assess the baseline risk and protective factors associated with mental health outcomes and will include 10 timepoints across the 27-month data collection period. The main objective of the study is to examine the underlying social drivers and mechanisms through which vocational training may impact mental health. The TOPOWA study will utilize surveys (self-reported measures), wearable fitness devices (to track sleep health, activities, and stress levels using heart rate variability), daily diaries (self-reported measures), neurological assessments (startle reflex), biomarkers (cortisol, dehydroepiandrosterone, alpha-amylase via saliva), and urine drug screens (to screen for benzodiazepines, barbiturates, methadone, propoxyphene, cannabinoids, cocaine, amphetamines, methaqualone, ecstasy, opiates, in addition to adulterants and alcohol). 

Surveys will be conducted at each timepoint. Abbreviated surveys will be used for intermediate timepoints, with more extensive surveys conducted at the baseline, post-intervention, and last time point. Survey measures consist of items on mental health outcomes (depression, anxiety, substance use, and suicidal ideation and attempts), risk factors (e.g., gender-based violence, childhood adversity, sexually transmitted infections, and discrimination), and protective factors (e.g., social support, family living status, household economic support). 

### 2.3. Measures

All measures were obtained from the baseline surveys across the two pilot studies. Demographic questions consisted of age, education, number of children, employment status, and living situation. Food security measures consisted of asking whether members of the household went to sleep hungry in the last month or if there was no food in the household at some point in the past month. Economic support (e.g., cash transfers, material support for education, food assistance, social pension) was measured using, “Has your household received any form of external financial support in the past 12 months?”.

The Kessler Psychological Distress Scale (K6) is typically used to measure global psychological distress and consists of six questions on the frequency of symptoms in the past 30 days [22,23]. The K6 and K10 have been validated in South Africa, but more studies are needed to validate this scale for individuals in Uganda and in the Luganda language [23]. This study examined only depressive symptoms as obtained from a single question within the K6. Depressive symptoms were operationalized using one question and dichotomized because of the small sample size to “depressed half of the time or less in the past 30 days” and “depressed more than half of the time during the past 30 days”. The K6 was not used in its entirety due to missingness on some of the questions, leading to decreases in the already small sample size. 

Perceived stress was measured using the Perceived Stress Scale (PSS) [24]. The PSS consists of 10 questions about stressful situations or symptoms that occurred in the previous month. The PSS is then scored to produce a categorical variable to indicate low, moderate, and high average stress levels [25]. The PSS categorical variable was collapsed to compare high stress levels compared to low/moderate stress levels due to the small sample size. 

The Ugandan Youth Quality of Life (QOL) questionnaire was used to measure life satisfaction in domains such as friendships, family relationships, money and financial security, neighborhood cohesion, and access to health and transportation services [25]. Using only the life satisfaction-oriented questions, we created mean satisfaction scores (1–5 with 5 being very satisfied and 1 being very dissatisfied) for each of the three domains identified by the original authors of the scale: living conditions and lifestyle, social relationships, and personal independence. Living conditions and lifestyle included seven questions on satisfaction with clothing, food, sex life, personal safety, health services, access to transportation, and time. Social relationships included five questions on how the participant gets along with their friends, how satisfied they are with their number of friends, and how they get along with their family and people in the community. Personal independence included five questions on how satisfied the participant is with the amount of money they have, work opportunities available, time without worry, and the amount of control over their money. Each of the questions corresponded with a numeric score, and the mean of all responses was obtained among the number of completed questions in each domain for each participant. Missing responses were excluded from the total mean calculation. Overall life satisfaction was measured using a single ladder scale scored out of 10, with 1 being the worst possible life satisfaction and 10 being the best possible life satisfaction. 

No interventions were received prior to the administration of these surveys. All survey questions were translated and back-translated into Luganda, the local Uganda language. Participants could also complete the surveys in English. In these analyses, we use the findings from this baseline survey. 

### 2.4. Data Analysis

Descriptive statistics were computed for demographic variables, food insecurity, life satisfaction, perceived stress, and depressive symptoms. Bivariate associations were computed for (1) differences in demographic characteristics and economic support between those with frequent depressive symptoms compared to those with less frequent depressive symptoms, (2) differences in demographic characteristics and economic support between those with low/moderate compared to high perceived stress levels, and (3) determination of the QOL domains (lifestyle and living conditions, social relationships, and personal independence) associated with high stress levels and frequent depressive symptoms. Bivariate statistical tests for categorical variables included chi-square tests and Fisher’s exact tests (when expected cell counts were <5), while continuous variables were compared using independent sample *t*-tests for means. All assumptions of these statistical tests were satisfied. Statistical significance was set *a priori* at α = 0.05. No multiple testing corrections were applied to the results of the statistical tests. All analyses were conducted in R 4.2.2.

## 3. Results

Among the total sample of participants in the two pilot studies (n = 60), the majority of participants reported completing secondary education or higher (69.5%) (Table 1). The mean age of participants was 20 years old (standard deviation = 1.83). Only 26.7% of participants reported having a current job. The majority (60.0%) of women also had four or more people living in their household. Thirty-two percent of women had children. Almost half (49.2%) of the women reported that someone in their household went to sleep hungry in the past 4 weeks. Additionally, 30.5% of women reported receiving household economic support at some time in the past 12 months. 

Nearly a quarter of participants (23.3%) reported high perceived stress levels, while 76.7% reported low or moderate stress levels (Table 2). No statistically significant differences were found in demographic variables, economic factors, and food insecurity between perceived stress levels. 

Among the participants who completed the depression question (n = 59), the majority of participants (67.8%) reported being depressed half of the time or less, including no depression, leaving 32.2% who reported being depressed most or all of the time in the past 30 days. Among those who reported high perceived stress levels (n = 14), 50% reported being depressed most or all of the time. However, among those with lower stress levels who also responded to the depression question (n = 45), 26.7% reported being depressed most or all of the time. 

A higher percentage of participants who reported depression most or all of the time also reported going to sleep hungry at some point in the past month (73.7%) compared to those reporting less frequent or no depression (37.5%, $\chi^{2}$ = 5.38, *df* = 1, *p* = 0.02). The majority of participants with more frequent depressive symptoms also reported having no food at some point in the past month compared to those with less frequent depressive symptoms (68.4% vs. 40.0%, respectively, $\chi^{2}$ = 3.10, *df* = 1, *p* = 0.08). However, these findings were not statistically significant. Additionally, those with more frequent depressive symptoms had a higher percentage of reporting receiving economic support in the past 12 months; however, this was not statistically significant (42.1% vs. 25.0%, Fisher’s exact test *p* = 0.23). No statistically significant differences were found in demographics or food insecurity between participants recording high vs low/moderate perceived stress. 

Life satisfaction, perceived stress, and depressive symptoms are displayed in Table 3. Participants who reported high stress levels had significantly lower life satisfaction scores in all three domains (living conditions and lifestyle, social relationships, and personal independence) compared to those with low/moderate stress. Overall life satisfaction scores (range 1–10) were 2 points lower for those with high stress levels compared to those with low/moderate stress levels (2.60 vs. 4.68, *t* = 2.74, *df* = 6.20, *p* = 0.03). 

While all measured aspects of life satisfaction had lower means for those who reported being depressed most or all of the time, only scores for the living conditions and lifestyle domain were significantly lower among those who reported being depressed most or all of the time (2.40) compared to those with no depression or depressive symptoms half of the time or less (2.74; *t* = 2.22, *df* = 36.18, *p* = 0.03). The other domain scores (social relationships and personal independence) and overall life satisfaction scores were not statistically significantly different between depression categories. 

## 4. Discussion

In this small study of women living in the slums of Kampala, nearly a quarter of 60 participants reported high stress levels and about one third of participants had depressive symptoms most or all of the past 30 days. This is consistent with previous research on depressive symptoms, which showed a 30% overall prevalence of depression among adults in Uganda [11]. Moreover, while demographics and economic factors (e.g., employment, education, external support) were not associated with high stress levels, the prevalence of household food insecurity was associated with more frequent depressive symptoms among participants. Previous studies have shown a link between food insecurity, stress, and mental health [3,26], which follows the SDoMH and MHN theoretical frameworks. While stress and depression often co-occur, depression may be more tied to food insecurity than stress is in our sample. 

Furthermore, factors associated with food insecurity may also influence stress levels, including parenting responsibilities for multiple children, lack of social and familial support, inadequate work opportunities, and inadequate food supply and access associated with climate change [26,27,28]. Future research should examine the interrelationships of these factors among women in low-resource settings and particularly in the urban environment. Emerging mental health research [29,30] also underscores the importance of assessing environmental factors including climate change in urban areas, which we did not address in our pilot studies. Women may also be disproportionately affected by adverse mental health outcomes related to climate change compared to men [29]. For example, excessive heat related to climate change can adversely affect pregnant women and reproductive outcomes [31]. Floods have also been a concerning issue associated with displacement and sleep disturbance, anxiety, stress, and increased alcohol use in specific areas within sub-Saharan Africa [32].

Life satisfaction scores were overall lower for women who reported high stress levels compared to those reporting low/moderate stress levels. Compared to previous work with Ugandan youth, participants in the current study reported lower life satisfaction scores [25]. As such, life satisfaction domains may shed new light on modifiable risk factors that may affect stress and ultimately other mental health outcomes. The living conditions and lifestyle domain (which includes satisfaction associated with a range of factors including clothing, food, sex life, personal safety, health services, access to transportation, and the way time is spent) scored significantly lower for women with high stress levels (compared to low/moderate stress) and for women with more frequent depressive symptoms. Many of the young women in the pilot study reported having children, which may exacerbate stress levels for those who do not have their basic needs met, including adequate access to health services, food, and transportation. Future work should examine these family structures and responsibilities related to children (including how many children they support). In addition, housing insecurity and a lack of infrastructure in the slums may also play a significant role in stress related to living conditions and lifestyle [33]. 

While no statistically significant differences in stress levels were noted for the number of people living in the same household, women with high stress levels also reported a higher percentage of three people living in their household compared to women with low/moderate stress levels. This may reflect an underlying burden on the young women who may have to support multiple generations, including their own children and aging parents. Women’s roles in household dynamics are also an important factor in examining the social drivers for mental health. In addition to assessing the number of children and people living in the household, it is imperative to collect more information on the household dynamics and women’s empowerment within their familial structures. 

Consistent with previous research on life satisfaction among youth living in Uganda [25], our findings showed that women who reported higher stress levels were significantly more likely to report lower satisfaction with social relationships. The complexities of the family structure and dynamics are important to evaluate alongside mental health outcomes such as stress and depression. Women reporting lower satisfaction scores with social relationships may have escaped complicated family dynamics such as abuse, child labor, and poverty, which is specific to the study population. In previous work, reporting a married partner was positively associated with higher quality of life scores [25]. Other measures on social relationships such as the quality of the relationship are needed to truly assess the drivers associated with higher stress levels and poor mental health. 

While we did not collect information on the types of healthcare services needed and barriers to accessing such services, these factors may represent optimal intervention targets for stress and depression (alongside physical health) needs. Given the unmet mental health needs among the women in this study, increased service provision is critically important. A task-sharing approach to mental health treatment might prove beneficial in this and other low-resource settings. For example, an intervention examining non-specialist health providers delivering mental health services was deemed to be feasible and acceptable across five low and middle-income country (LMIC) sites, including Uganda [34]. YouBelong Home is another community mental health program which may be beneficial in increasing access to mental health care for Ugandan women [35]. Furthermore, it will be important for new interventions to incorporate strategies to overcome gender-specific adversities, such as exposure to gender-based violence (GBV). Satisfaction with these interventions and improved mental and physical healthcare should also be measured alongside intervention effectiveness. 

Addressing the social drivers directly may also affect downstream mental health adversities. Economic support and gender-empowerment initiatives have the potential to shift cultural norms and reduce gender-based violence associated with the unequal distribution of resources. While this study did not specifically examine gender-based violence, this analysis found that high stress was associated with dissatisfaction with personal independence and social relationships, which can be impacted by gender-based violence. Previous work has shown promising results for addressing gender-based violence in low-resource settings [36]. This highlights the need to address the multi-faceted approach to gender-based violence and corresponding mental health outcomes. 

### Limitations

While the findings from this study show important potential intervention targets for mental health concerns among young women, there are several limitations that should be considered when interpreting the results. First, the sample size from this study is limited due to the primary intent of the feasibility assessment for the data collection in the two pilot studies. Because of the small sample size and occasional missing data, this study examined a single question from the K6 and a categorical variable obtained from the PSS score calculation. No statistical tests had multiple testing corrections applied, meaning that several associations may be statistically significant simply due to chance. The analyses were not able to assess the social drivers for mental health in a multivariable setting due to the small sample size. Additionally, factor analysis was not able to be conducted on the Uganda Quality of Life index because of the sample size, which would be beneficial for examining the different domains of life satisfaction in this population, including addressing importance scores and computing population-specific weights. Therefore, the replication of these findings is necessary prior to the development or modification of interventions for this population. The planned cohort study will support this need and its baseline survey will be adequately powered to assess findings of the full K6 and PSS. The participants may not be representative of all women living in the slums of Kampala because these women were recruited through specific centers which are part of the UYDEL organization, which provides vocational training and psychosocial support. Therefore, this study sample may be more reflective of a subgroup of women who are highly motivated to seek services within the broader population of women in the slums. Social desirability and recall bias may also have affected our results relating to sensitive topics on the satisfaction of sex life, economic hardships, and financial security. Lastly, specific questions on healthcare access (within the lifestyle and living conditions domain) did not differentiate between physical and mental health, which may greatly inform targeted interventions. 

## 5. Conclusions

Despite these limitations, this analysis presents important findings related to preliminary data for social drivers of mental health and mental health adversities experienced by young women and girls living in the slums of Kampala. This study found that 23.3% of young women living in the slums of Kampala, Uganda, reported high stress levels and 32.2% reported depressive symptoms on the majority of days. Additionally, high stress levels were associated with a dissatisfaction with the lifestyle and living conditions domain (clothing, access to health services and transportation, the way they spend their time, and the amount of money and job opportunities). Frequent depressive symptoms were associated with being dissatisfied with life overall, including all three domains (lifestyle and living conditions, social relationships, and personal independence). These findings are intended to inform targets for mental health interventions among women in low-resource urban settings and to generate interest in research that examines the complex associations between social drivers and mental health outcomes among women in LMICs. These initial findings represent an early dissemination of research prior to replication in the larger TOPOWA cohort study, where many of the limitations noted in this analysis will be addressed. 

## Figures and Tables

**Table 1:** Demographic characteristics and economic insecurity of TOPOWA Project pilot women study participants ages 18 to 24 years of age living in the urban slums of Kampala by perceived stress levels (n = 60).

	Low/Moderate Perceived Stress (n = 46) 76.7%	High Perceived Stress (n = 14) 23.3%	Overall (n = 60)	Test Statistic, *df*, *p*-Value
Age, Mean (SD)	20.13 (1.92)	20.07 (1.59)	20.12 (1.83)	*t* = 0.12, *df* = 25.56, *p* = 0.91
Do you have children?				Fisher’s Exact Test *p* = 0.75
Yes	14 (30.4%)	5 (35.7%)	19 (31.7%)	
No	32 (69.6%)	9 (64.3%)	41 (68.3%)	
Highest level of education *				Fisher’s Exact Test *p* = 0.12
Completed some primary/all primary	16 (34.8%)	2 (14.3%)	18 (30.0%)	
Completed secondary or higher	30 (65.2%)	12 (85.7%)	42 (70.0%)	
Do you have a job?				Fisher’s Exact Test *p* = 0.74
Yes	13 (28.3%)	3 (21.4%)	16 (26.7%)	
No	33 (71.7%)	11 (78.6%)	44 (73.3%)	
How many people are living in your household?				Fisher’s Exact Test *p* = 0.16
Two	11 (23.9%)	1 (7.1%)	12 (20.0%)	
Three	7 (15.2%)	5 (35.7%)	12 (20.0%)	
Four or more	28 (60.9%)	8 (57.1%)	36 (60.0%)	
Did you or any household member go to sleep at night hungry because there was not enough food? (past month)				Fisher’s Exact Test *p* = 0.76
Yes	24 (52.2%)	6 (42.9%)	30 (50.0%)	
No	22 (47.8%)	8 (57.1%)	30 (50.0%)	
Was there ever no food to eat of any kind in your household because of lack of resources to get food? (past month)				Fisher’s Exact Test *p* = 0.76
Yes	24 (52.2%)	6 (42.9%)	30 (50.0%)	
No	22 (47.8%)	8 (57.1%)	30 (50.0%)	
Has your household received any form of external economic support in the past 12 months?				Fisher’s Exact Test *p* = 1.00
Yes	14 (30.4%)	4 (28.6%)	18 (30.0%)	
No	32 (69.6%)	10 (71.4%)	42 (70.0%)	

* = Cells collapsed for small sample sizes and protection of anonymity. *df* = degrees of freedom.

**Table 2:** Demographic characteristics and economic insecurity of TOPOWA Project pilot women study participants ages 18 to 24 years of age living in the urban slums of Kampala by the presence of self-reported depressive symptoms (n = 59).

	Depressed Half of the Time or Less in the Past 30 Days (n = 40) 67.8%	Depressed Most or All of the Time in the Past 30 Days (n = 19) 32.2%	Overall (n = 59)	Test Statistic, *df*, *p*-Value
Age, Mean (SD)	20.13 (1.88)	20.16 (1.80)	20.12 (1.83)	*t* = −0.06, *df = 36.92*, *p* = 0.95
Do you have children?				Fisher’s Exact Test *p* = 0.76
Yes	12 (30.0%)	7 (36.8%)	19 (32.2%)	
No	28 (70.0%)	12 (63.2%)	40 (67.8%)	
Highest level of education *				Fisher’s Exact Test *p* = 0.55
Completed some primary/all primary	11 (27.5%)	7 (36.8%)	18 (30.5%)	
Completed secondary or higher	29 (72.5%)	12 (63.2%)	41 (69.5%)	
Do you have a job?				Fisher’s Exact Test *p* = 0.76
Yes	10 (25.0%)	6 (31.6%)	16 (27.1%)	
No	30 (75.0%)	13 (68.4%)	43 (72.9%)	
How many people are living in your household?				Fisher’s Exact Test *p* = 1.00
Two	8 (20.0%)	3 (15.8%)	11 (18.6%)	
Three	8 (20.0%)	4 (21.1%)	12 (20.3%)	
Four or more	24 (60.0%)	12 (63.2%)	36 (61.0%)	
Did you or any household member go to sleep at night hungry because there was not enough food? (past month)				$\chi^{2}$ = 5.38, *df* = 1, ***p* = 0.02**
Yes	15 (37.5%)	14 (73.7%)	29 (49.2%)	
No	25 (62.5%)	5 (26.3%)	30 (50.8%)	
Was there ever no food to eat of any kind in your household because of lack of resources to get food? (past month)				$\chi^{2}$ = 3.10, *df* = 1, *p* = 0.08
Yes	16 (40.0%)	13 (68.4%)	29 (49.2%)	
No	24 (60.0%)	6 (31.6%)	30 (50.8%)	
Has your household received any form of external economic support in the past 12 months?				Fisher’s Exact Test *p* = 0.23
Yes	10 (25.0%)	8 (42.1%)	18 (30.5%)	
No	30 (75.0%)	11 (57.9%)	41 (69.5%)	

Note. One participant had a missing response and is not included in table. * = Cells collapsed for small sample sizes and protection of anonymity. *df* = degrees of freedom. Statistically significant associations are bolded.

**Table 3:** Life satisfaction, perceived stress, and depressive symptoms among TOPOWA Project pilot women study participants ages 18 to 24 years of age living in the urban slums of Kampala, (n = 60).

	**Low/Moderate Perceived Stress** **n = 46 (76.7%)**	**High Perceived Stress** **n = 14 (23.3%)**	**Test Statistic, df, *p*-Value**
	**Mean (SD)**	**Mean (SD)**	
Life Satisfaction: Living conditions and lifestyle	2.76 (0.55)	2.15 (0.39)	*t* = 4.57, *df* = 30.22, ***p* < 0.001**
Life Satisfaction: Social relationships	3.33 (0.66)	2.73 (0.60)	*t* = 3.22, *df* = 23.85, ***p* = 0.004**
Life Satisfaction: Personal independence	2.76 (0.77)	2.27 (0.24)	*t* = 3.66, *df* = 55.98, ***p* < 0.001**
Overall, how satisfied are you with your life?	4.68 (1.70)	2.60 (1.52)	*t* = 2.74, *df* = 6.20, ***p* = 0.03**
	**Depressed Half of the Time or Less Including no Depression** **n = 40 (67.8%)**	**Depressed Most or All of the Time** **n = 19 (32.2%)**	**Test Statistic, *df*, *p*-Value**
	**Mean (SD)**	**Mean (SD)**	
Life Satisfaction: Living conditions and lifestyle	2.74 (0.56)	2.40 (0.54)	*t* = 2.22, *df* = 36.18, ***p* = 0.03**
Life Satisfaction: Social relationships	3.24 (0.65)	3.07 (0.79)	*t* = 0.80, *df* = 30.41, *p* = 0.43
Life Satisfaction: Personal independence	2.69 (0.77)	2.58 (0.59)	*t* = 0.59, *df* = 42.53, *p* = 0.56
Overall, how satisfied are you with your life?	4.88 (1.50)	3.71 (2.02)	*t* = 1.77, *df* = 23.80, *p* = 0.09

Note. Depressive symptoms were assessed for the past month. One participant had a missing response on the depression question. *p*-values corresponding to statistically significant differences are bolded.

## Data Availability

Data requests can be made directly to the PI/corresponding author.
